# Internet access and use by COPD patients in the National Emphysema/COPD Association Survey

**DOI:** 10.1186/1471-2466-14-66

**Published:** 2014-04-22

**Authors:** Carlos H Martinez, Beth L St Jean, Craig A Plauschinat, Barbara Rogers, Julen Beresford, Fernando J Martinez, Caroline R Richardson, MeiLan K Han

**Affiliations:** 1 Division of Pulmonary and Critical Care, University of Michigan Health System, 3916 Tubman Center, 1500 E. Medical Center Drive, Box 0360, Ann Arbor, USA; 2 College of Information Studies, University of Maryland, College Park, MD, USA; 3 Novartis Pharmaceutical Corporation, East Hanover, NJ, USA; 4 National Emphysema/COPD Association, New York, NY, USA; 5 Department of Family Medicine, University of Michigan Health System, 1018 Fuller St., 48104-1213 Ann Arbor, MI, USA; 6 Ann Arbor VA Center for Clinical Management Research, Ann Arbor VA Healthcare System, 2215 Fuller Rd, 48105 Ann Arbor, MI, USA

**Keywords:** Chronic obstructive pulmonary disease, Internet, Elderly adults, Chronic disease management, Multimorbidity, Health information seeking, Digital divide

## Abstract

**Background:**

Technology offers opportunities to improve healthcare, but little is known about Internet use by COPD patients. We tested two hypotheses: Internet access is associated with socio-demographic disparities and frequency of use is related to perceived needs.

**Methods:**

We analyzed data from a 2007–2008 national convenience sample survey of COPD patients to determine the relationship between Internet access and frequency of use with demographics, socio-economic status, COPD severity, and satisfaction with healthcare.

**Results:**

Among survey respondents (response rate 7.2%; n = 914, 59.1% women, mean age 71.2 years), 34.2% reported lack of Internet access, and an additional 49% had access but used the Internet less than weekly. Multivariate models showed association between lack of access and older age (OR 1.10, 95% CI 1.07, 1.13), lower income (income below $30,000 OR 2.47, 95% CI 1.63, 3.73), less education (high school highest attainment OR 2.30, 95% CI 1.54, 3.45), comorbid arthritis or mobility-related disease (OR 1.56, 95% CI 1.05, 2.34). More frequent use (at least weekly) was associated with younger age (OR 0.95, 95% CI 0.93, 0.98), absence of cardiovascular disease (OR 0.48, 95% CI 0.29, 0.78), but with perception of needs insufficiently met by the healthcare system, including diagnostic delay (OR 1.72, 95% CI 1.06, 2.78), feeling treated poorly (OR 2.46, 95% CI 1.15, 5.24), insufficient physician time (OR 2.29, 95% CI 1.02, 5.13), and feeling their physician did not listen (OR 3.14, 95% CI 1.42, 6.95).

**Conclusions:**

An analysis of the characteristics associated with Internet access and use among COPD patients identified two different patient populations. Lack of Internet access was a marker of socioeconomic disparity and mobility-associated diseases, while frequent Internet use was associated with less somatic disease but dissatisfaction with care.

## Background

The burden of chronic illness in our society is significant; almost half of Americans have at least one chronic disease. This percentage rises to 80% among Medicare beneficiaries, with 23% of this population reporting five or more chronic concurrent diseases [1,2]. The negative effects of chronic illness include functional impairment, limited mobility, and poorer quality-of-life (QOL) [3]. Chronic obstructive pulmonary disease (COPD) patients, afflicted by the third leading cause of mortality [4], face significant challenges in accessing healthcare services, due to the presence of comorbidities [5], limited mobility [6], advanced age, the effects of social isolation, and often rural residency [7]. All of these factors create additional needs, many of which go unmet by the healthcare system. To address these needs, innovative strategies, particularly those utilizing the Internet have emerged. Internet-mediated strategies for dyspnea management [8], smoking cessation [9], and exercise and physical activity [10] have been tested or implemented. Internet-mediated programs, however, will only benefit patients who have access to and who use the Internet. Recent surveys tracking the penetration of new communication technologies among the chronically ill [11] show that different diseases are associated with varying levels of information resource use [12].

**Figure 1:**
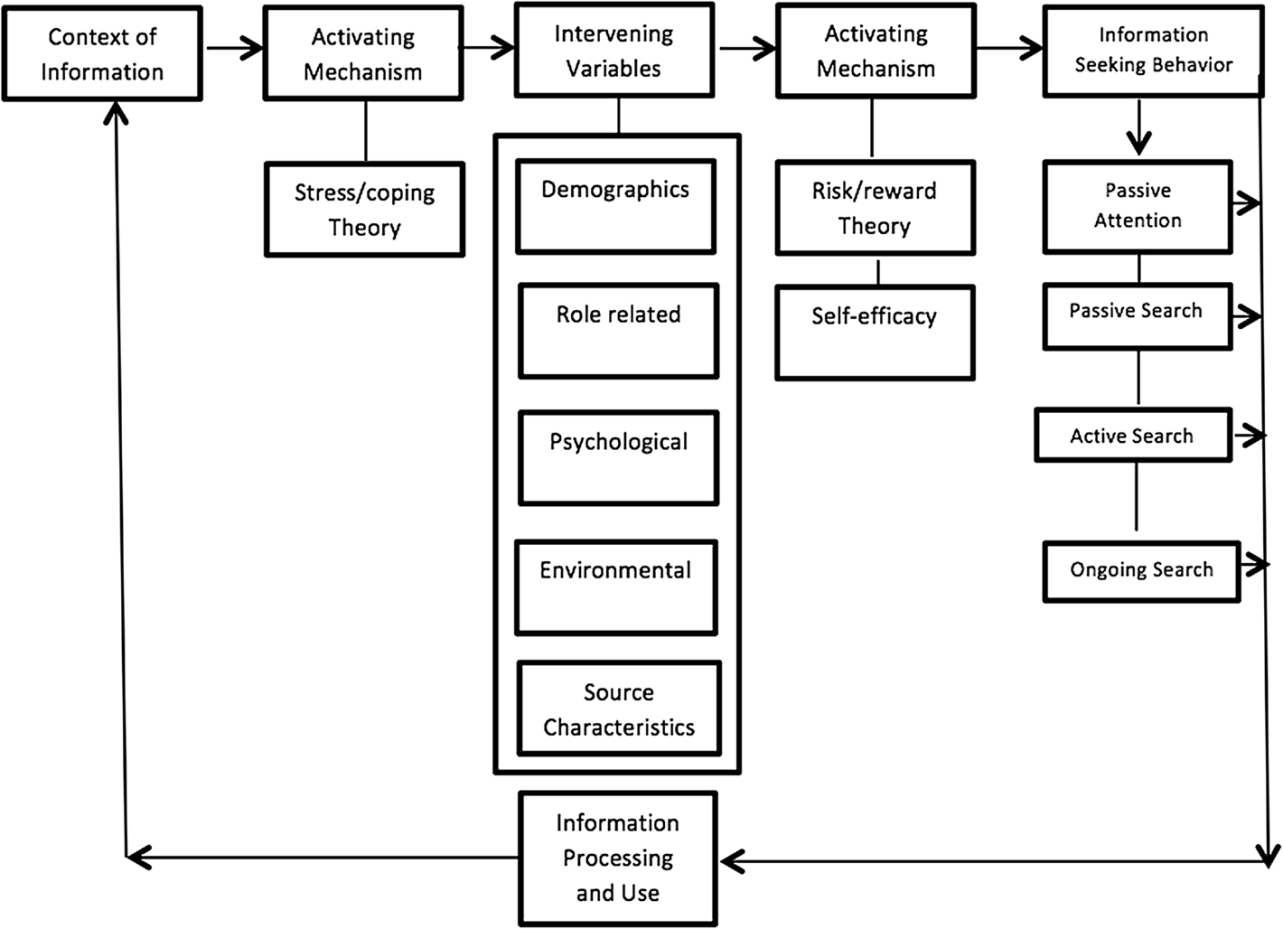
**Modified Wilson’s 1996 Model of Information Behavior.** This model depicts that a person develops an information need due to some basic stressor (a new diagnosis or disease status), and that intervening variables (demographics and socioeconomic variables) then motivate or impede the person’s information seeking and use. Additional mechanisms based on the perceived risks and rewards (termed here “perceived needs”) further influence patients’ decisions regarding whether and how actively to seek information and what information sources to consult (from a passive diffusion of information to an active ongoing search). Following information seeking behavior, a feedback loop is created, based upon the actions taken (or not) to process and use the information (a step not modeled here).

**Figure 2:**
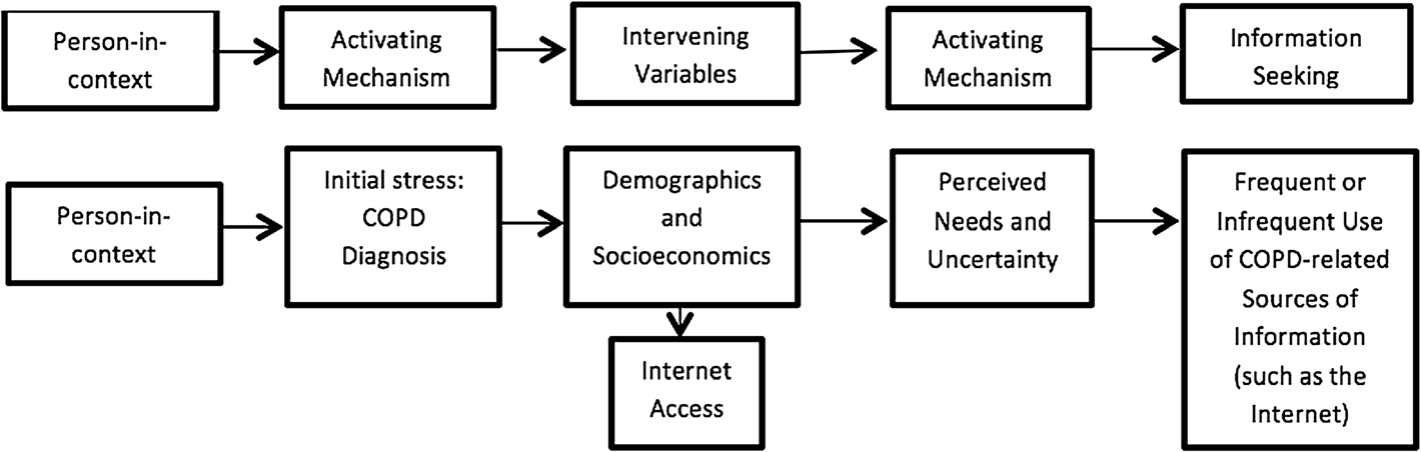
**Modified Wilson’s 1996 Model of Information Behavior applied to COPD.** The modified and simplified model depicts that a person develops an information need due to the stressor of having a COPD diagnosis, and that demographics and socioeconomic variables (intervening variables) define the access to the information source (Internet). After that, perceived and unmet needs (risks and rewards) further influence patients’ decisions regarding frequency of Internet use. A final feedback loop describing the actions taken (or not) is not modeled here. The current project tested this theoretical model.

In response to the limited availability of data on COPD patients’ Internet access and use [13], we used data from a national survey of COPD patients to evaluate the relationship between patients’ socioeconomic factors, clinical characteristics, comorbidities, disease severity, and their reported Internet access and frequency of use. Based on a modified framework of Wilson’s 1996 Model of Information Behavior (Figures 1 and 2) and other models of information-seeking behavior in the chronically ill patients used in large information survey design [14], we developed a model of Internet access and use among COPD patients. We predicted that personal characteristics can precede the ability to access a service (building of awareness or pre-contemplation), and that ensuing needs influence the patient’s decision to seek information (awareness and action). The current analysis tests two hypotheses: (1) Internet access is determined by demographic and socioeconomic factors, and will be less common among disadvantaged patient populations and (2) among patients with Internet access, frequency of use is related to perceived health needs.

## Methods

The current analysis uses data from a national survey of patients with COPD, which was commissioned by the National Emphysema/COPD Association (NECA) and conducted from the fall of 2007 to winter of 2008.

### Patient survey

The methods of the NECA-commissioned national surveys of COPD patients have been detailed previously [15]. Briefly, participants in the survey were randomly selected from either one of four different sources, all of them sampled during the same period of time: (1) a national sample of households in which at least one person reported a diagnosis of COPD based on a database maintained by Integrated Business Services Inc. (Lake Forest, IL); (2) participants of patient support groups affiliated with the American Lung Association’s Better Breathers Clubs or NECA; (3) COPD patients receiving oxygen through a national provider (Apria Healthcare, Lake Forest, CA); and (4) respondents to an Internet survey invitation on COPD-related websites. All participants were mailed a 7-page questionnaire, accompanied by an explanatory letter and a postage-paid return envelope. Households not willing to participate in the survey, according to response to previous contacts, were excluded. The incentive for participation was an executive summary of the survey results. No economic incentives were offered. The total number of completed surveys was 1,077 out of 15,000 surveys sent (response rate 7.2%). For the purpose of the current analyses, people who reported a diagnosis of asthma or alpha-1 anti-trypsin deficiency but not COPD were excluded, leaving 914 COPD patients for analysis.

### Questionnaire contents and independent variables

The content of the questionnaire was prepared with the help of COPD expert panels, including physicians, patients, and representatives of COPD organizations. The items were considered to have face validity and were easy to understand, and have been used in prior research efforts [15]. Questions included demographics, comorbid conditions, perceived health and limitations for daily activities, use of health-care resources including visits to physicians, and sources of information consulted about the disease. Annual household income was selected from a list of options, and recoded pooling the answers in two groups–below $30,000 and $30,000 and above. Comorbid conditions were self-reported, based on a list of conditions presented. Disease severity and impact were evaluated by the Medical Research Council (MRC) Dyspnea Index to describe severity of breathlessness [16]. The living with COPD (LCOPD) Questionnaire, a patient-reported outcome measure, was also used to evaluate the overall impact of disease. The LCOPD instrument is a 22-item scale, with scores ranging between 0–22 [17], higher scores represent poorer QOL. The frequency of visits to any doctor and pulmonologist during the prior 12 months was also evaluated. Multiple questions addressed patients’ perceptions of their needs and experiences with the healthcare system, including their level of satisfaction with their physician, medications, amount of time spent with their physician, ability to reach their physician and make appointments without delay, and their perception of how they were treated by the medical community in general. Participants also had the opportunity to select from a list of all other sources of education and information regarding their disease that they had regularly used. The full questionnaire is available as Additional file 1.

Based on our *a priori* hypotheses, we created sets of independent variables to be tested as potential explanatory factors, including personal characteristics (i.e., age, gender, education, income, rurality, and insurance), disease severity (i.e., symptoms, dyspnea severity according to the MRC Dyspnea Index, exacerbations, oxygen use, continuous oral steroid use, and QOL score), comorbidities, perceived needs (i.e., severity of symptoms, work limitations, and experience and satisfaction with the healthcare system and with healthcare providers). All of these variables were tested for their relation with the outcomes of interest, Internet access and frequency of use.

### Outcomes: Internet access and frequency of use

An additional specific question about Internet use (“How often do you use the Internet to get information about your condition or its treatment?”) was included, with options ranging from “at least weekly” to “only a few times per year, rarely/never”, and a final option for “no Internet access”. Based on participants’ responses to this question, two different categories were created: Internet access and frequency of Internet use. Participants who responded “no Internet access” were compared with all others (participants with access), independently of frequency of use. To evaluate potential determinants of frequency of use, the respondents with no access were excluded, and the participants with “at least weekly” use were compared with the rest of the population with Internet access.

### Statistical analysis

Data are presented as proportions and means, with standard deviations (SDs) as appropriate. Statistical comparisons for categorical data were made using chi-square tests. Continuous data was compared using t-tests. We analyzed the bivariate relationships between both “lack of Internet access” or “frequent Internet use” and the sets of explanatory variables. Separate logistic models with the outcomes “lack of access” and “frequent use” were constructed, with inclusion of all sets of pre-specified explanatory variables. All p-values were 2-tailed, with a p-value less than 0.05 considered statistically significant. Analyses were performed using SAS 9.2 (SAS Institute, Cary, NC).

### Ethical aspects and role of the funding agencies

The current project uses de-identified data from a survey with no potential links to any subject identifier. The project was approved by the University of Michigan Medical School Institutional Review Board (Study ID HUM00076780). Novartis provided funding to conduct the survey but was not involved in the data analysis. NECA and Novartis participated in the review and approval of this manuscript. The database containing the raw survey data is maintained by Novartis and Innovative Health Solutions.

## Results

### Bivariate relationships: internet access

The demographic characteristics of the 914 COPD patients who responded to the survey (response rate 7.2%), along with variables relating to their disease severity and disease management, are shown in Table 1 stratified by Internet access and frequency of Internet use. Table 2 lists participants’ comorbidities and perceived needs, also stratified by Internet access and frequency of Internet use.

**Table 1:** Demographic, socioeconomic, and clinical characteristics of patients by internet access and frequency of internet use

	**Internet access**			**Frequency of internet use**		
	**Yes N = 590**	**No N = 324**	**p-value**	**At least weekly N = 150**	**Less than weekly N = 440**	**p-value**
**Demographics**						
Mean age	69.0 (9.8)	75.1 (7.3)	< 0.0001	62.8 (10.0)	71.1 (8.8)	< 0.0001
Female gender	56.95	62.96	0.07	67.33	53.41	0.003
Education HS or Lower	33.45	57.59	< 0.0001	34.00	33.26	0.92
Income < $30,000	41.11	68.18	< 0.0001	49.66	37.85	0.01
Living in rural area	22.32	26.42	0.19	31.33	19.22	0.003
White race	96.10	96.54	0.10	97.33	95.67	0.10
**Insurance (%)**						
Medicare	71.69	81.23	0.001	57.62	76.54	< 0.0001
Employer/work	33.05	26.10	0.026	30.46	33.94	0.43
None	2.37	1.77	0.54	5.30	1.37	0.006
**Disease severity (%)**						
**QOL score**	10.1 (6.1)	9.4 (6.0)	0.10	12.6 (5.8)	9.2 (6.0)	< 0.0001
**Symptoms**						
Shortness of breath	84.09	80.94	0.21	88.74	82.50	0.07
Cough	55.67	58.65	0.37	54.97	55.91	0.84
Phlegm	55.50	54.84	0.84	55.63	55.45	0.97
Nocturnal symptoms	35.53	35.78	0.94	38.41	34.55	0.39
Any exacerbation	43.99	41.64	0.48	58.28	39.09	< 0.0001
At least one hospital admission	25.72	27.86	0.47	29.80	24.32	0.18
At least one emergency room visit	26.40	24.05	0.42	35.76	23.18	0.0025
Severe dyspnea	24.96	26.20	0.74	29.14	23.49	0.20
**Disease management**						
Oxygen use	60.52	58.66	0.58	75.50	55.24	< 0.0001
Continuous oral steroids	18.80	16.61	0.47	21.48	17.86	0.39
**Self-reported compliance**						
Oxygen	84.53	73.03	0.001	87.72	82.98	0.25
Ever stopped meds	29.72	22.32	0.01	32.00	28.91	0.47
Low med compliance	13.04	19.33	0.01	13.42	12.90	0.87
Low persist meds	4.13	7.29	0.04	5.30	3.72	0.40

**Table 2:** Patients’ comorbidities and perceived needs by internet access and frequency of internet use

	**Internet access**			**Frequency of internet use**		
	**Yes N = 590**	**No N = 324**	**p-value**	**At least weekly N = 150**	**Less than weekly N = 440**	**p-value**
**Comorbidities (%)**						
Hypertension	44.58	53.37	0.009	28.48	50.11	< 0.0001
Heart disease	23.39	33.43	0.0009	17.88	25.28	0.06
Arthritis	35.93	53.08	< 0.0001	27.81	38.72	0.015
Osteoporosis	22.03	28.74	0.02	25.17	20.96	0.28
Diabetes	17.46	24.63	0.008	11.92	19.36	0.037
Anxiety	22.71	21.11	0.57	35.10	18.45	< 0.0001
Depression	22.71	20.82	0.50	32.45	19.36	0.0009
Obesity	21.19	15.84	0.045	31.79	17.54	0.0002
Sleep disorder	23.56	13.78	0.0003	22.52	23.92	0.72
Migraine	5.76	4.11	0.27	5.96	5.69	0.90
Mean comorbidities	2.7 (1.8)	3.0 (1.9)	0.01	2.8 (1.8)	2.7 (1.9)	0.80
**Disease by systems**						
Any psych disease	32.49	31.38	0.72	45.70	27.95	< 0.0001
Any cardiovasc disease	61.59	72.43	0.0008	44.37	67.50	< 0.0001
Any articular disease	46.19	62.76	< 0.0001	41.72	47.73	0.20
**Perceived needs (%)**						
Insufficient time with physician	10.00	7.28	0.21	20.53	6.29	< 0.0001
Dissatisfied with my physician	10.26	6.33	0.058	14.67	8.74	0.03
Diagnosis was delayed	39.42	19.35	< 0.0001	57.62	33.18	< 0.0001
Doctor not sympathetic	3.77	1.77	0.06	6.62	2.78	0.04
Doctor does not listen	8.92	10.14	0.56	17.22	5.94	< 0.0001
Treated poorly by healthcare system	10.28	14.02	0.11	23.33	5.19	< 0.0001
Doctor difficult to make appointment with	8.02	7.25	0.67	12.58	6.44	0.01
Doctor difficult to reach	12,84	12.10	0.74	20.53	10.16	0.001
Dissatisfied with meds	9.01	8.18	0.66	13.33	7.49	0.03
Poor/very poor health	22.75	17.40	0.06	33.77	18.95	0.0002
Severe/very severe symptoms	39.11	28.27	0.001	54.97	33.56	< 0.0001
Symptoms keep me from working	30.63	22.58	0.008	53.64	22.73	< 0.0001
Symptoms limit amount of work	48.56	45.75	0.40	56.95	45.68	0.016
Visits any doctor	8.0 (6.4)	6.7 (5.9)	0.002	9.1 (6.6)	7.6 (6.2)	0.015
Visits pulmonologist	3.0 (3.5)	2.4 (2.7)	0.010	3.9 (4.9)	2.7 (2.7)	0.002

Compared with those with Internet access, participants without Internet access were older (no access: mean age ± s.d. 75.1 ± 7.3 years; Internet access: 69.0 ± 9.8; p < 0.0001), less educated (high school as highest level of educational attainment 57.6% vs. 33.5%, p < 0.0001), and had lower income ($30,000 or below 68.2% vs. 41.1%, p < 0.0001). Furthermore, they were more frequently Medicare-insured (81.2% vs. 71.7%, p = 0.001), and less frequently employer-insured (26.1% vs. 33.1%, p = 0.02). No gender or ethnicity-related differences were found between persons with vs. without Internet access, and the descriptors of disease severity and management were similar, including symptoms, oxygen and steroid use, and compliance with treatment.

When comparing comorbidities between participants according to Internet access, those without access more frequently reported hypertension (53.4% vs. 44.6%, p = 0.009), heart disease (33.4% vs. 23.4%, p = 0.0009), arthritis (53.1% vs. 35.9%, p < 0.0001), and diabetes (24.6% vs. 17.5%, p = 0.008), but less frequently reported sleep disorders (15.8% vs. 21.2%, p = 0.04) and obesity (13.8% vs. 23.6%, p = 0.0003). Participants without Internet access also less frequently reported a perceived diagnosis delay (19.4% vs. 39.4%, p < 0.0001). In all other respects, however, the perceived needs of participants without Internet access were similar to those of participants with Internet access.

### Bivariate relationships: frequency of internet use

A similar analysis restricted to the participants reporting that they had Internet access was conducted, comparing participants who use the Internet frequently (i.e., at least weekly) with the rest of the participants. More frequent users were younger (62.8 ± 10 vs. 71.1 ± 8.8 years of age, p < 0.0001), more likely female (67.3% vs. 53.4%, p = 0.003), had income below $30,000 (49.7% vs. 37.9%, p = 0.01) and less frequently relied on Medicare insurance (57.6% vs. 76.5%, p = 0.003).

Regarding disease severity and comorbidities, frequent Internet users tended to report at least one COPD-related exacerbation (58.3% vs. 39.1%, p < 0.0001), and oxygen use (75.5% vs. 55.2%), although other descriptors of symptoms and disease severity were similar. More frequent Internet users reported poorer QOL scores (mean ± s.d. points in the LCOPD: 12.6 ± 5.8 vs. 9.2 ± 6.0, p < 0.0001). Comorbidity profiles showed that more frequent Internet users reported a lower frequency of hypertension (28.5% vs. 50.1%, p < 0.0001), arthritis (27.8% vs. 38.7%, p = 0.01) and diabetes (11.9% vs. 19.4%, p = 0.03), but a higher frequency of anxiety (35.1% vs. 18.5%, p < 0.0001), depression (32.5% vs. 19.4%, p = 0.0009) and obesity (31.8% vs. 17.5%, p = 0.0002).

Participants who used the Internet more frequently reported more perceived needs, including dissatisfaction with their physician (14.7% vs. 8.7%, p = 0.03), feeling that their doctor was not sympathetic (6.6% vs. 2.8%, p = 0.04), not a good listener (17.2% vs. 5.9%, p < 0.0001), more difficult to reach (20.5% vs. 10.2%, p = 0.001), and difficult to make appointments with (12.6% vs. 6.4%, p = 0.01). They also were more apt to report believing that their diagnosis was delayed (57.6% vs. 33.2%, p < 0.0001), a feeling of dissatisfaction with their treatment (13.3% vs. 7.5%, p = 0.03), and feeling that they were treated poorly by the healthcare system (23.3% vs. 5.2%, p < 0.0001). Furthermore, they reported more visits to their pulmonologist (mean number of visits 3.9 ± 4.9 vs. 2.7 ± 2.7, p = 0.002) and to any physician (9.1 ± 6.6 vs. 7.6 ± 6.2 visits, p = 0.01) during the prior year. Table 3 shows participants’ use of other information sources on COPD. Both respondents without Internet access and those who use the Internet less often reported less frequent use of almost all other information sources, with the exception of television or cable, physicians, and pamphlets/brochures.

**Table 3:** Use of educational resources and self-reported disease knowledge by internet access and frequency of use

	**Internet access**			**Frequency of internet use**		
	**Yes N = 590**	**No N = 324**	**p-value**	**At least weekly N = 150**	**Less than weekly N = 440**	**p-value**
**Source of education/information (%)**						
Physician	87.29	82.99	0.07	84.77	88.15	0.28
Respiratory therapist	45.25	38.71	0.051	43.71	45.79	0.65
Nurse	38.64	30.79	0.01	40.40	38.04	0.60
Books, magazines	39.66	31.09	0.008	47.68	36.90	0.019
Other patients	19.15	8.50	< 0.0001	27.81	16.17	0.0017
Television or cable	14.58	13.20	0.55	11.26	15.72	0.18
Patient organizations	15.25	7.04	0.0002	26.49	11.39	< 0.0001
Pamphlets, brochures	43.39	36.36	0.03	39.74	44.65	0.29
Online support groups	23.73	0.88	< 0.0001	65.56	9.34	< 0.0001
Other internet sites	37.46	1.17	< 0.0001	72.19	25.51	< 0.0001
**COPD knowledge (%)**						
Not well-informed	16.52	12.42	0.09	15.89	16.74	0.80
Not aware of guidelines	51.48	56.73	0.13	45.33	53.65	0.07

### Multivariate models

Multivariate models of Internet access and frequent Internet use adjusted for gender, insurance, exacerbations, comorbidities, visits to physicians, and indicators of disease severity were tested (Table 4). These models confirmed that lack of Internet access is independently associated with specific demographic characteristics: age increment was associated with likelihood of lack of access (OR 1.10, 95% CI 1.07, 1.13), as well as low income (OR 2.47, 95% CI 1.63, 3.73), low educational level (OR 2.30, 95% CI 1.54, 3.45), and the presence of mobility-related comorbid diseases (OR 1.56, 95% CI 1.05, 2.34). The presence of cardiovascular disease was not associated with lack of access (OR 1.16, 95% CI 0.77, 1.76).

**Table 4:** **Multivariate models for lack of internet access and more frequent internet use**^**a**^

	**Lack of internet access**	**More frequent internet use**
	**OR (95% CI)** ***p-value***	**OR (95% CI)** ***p-value***
**Demographics and comorbidities**		
Age (per 1-year increment)	1.10 (1.07, 1.13) *< 0.0001*	0.95 (0.93, 0.98) *0.002*
Low income	2.47 (1.63, 3.73) *< 0.0001*	1.26 (0.77, 2.06) *0.36*
Education below high school	2.30 (1.54, 3.45) *< 0.0001*	0.70 (0.41, 1.19) *0.18*
Cardiovascular disease	1.16 (0.77, 1.76) *0.47*	0.48 (0.29, 0.78) *0.003*
Mobility-related disease	1.56 (1.05, 2.34) *0.03*	0.84 (0.51, 1.38) *0.48*
Home oxygen use	0.88 (0.58, 1.33) *0.54*	2.71 (1.49, 4.91) *0.001*
Exacerbations last year	1.0(0.65, 1.45) *0.99*	1.54 (0.90, 2.64) *0.11*
**Perceived needs**		
Diagnosis was delayed	0.52 (0.34, 0.81) *0.004*	1.72 (1.06, 2.78) *0.03*
Treated poorly by healthcare system	1.99 (1.04, 3.81) *0.03*	2.46 (1.15, 5.24) *0.02*
Insufficient time with doctor	0.84 (0.38, 1.85) *0.67*	2.29 (1.02, 5.13) *0.04*
Doctor does not listen	1.19 (0.59, 2.43) *0.62*	3.14 (1.42, 6.95) *0.005*
Doctor not sympathetic	0.94 (0.0.21, 4.23) *0.94*	0.58 (0.14, 2.36) *0.45*
Doctor difficult to reach	1.11 (0.58, 2.11) *0.75*	0.98 (0.47, 2.02) *0.94*
Doctor difficult to make appointment with	1.28 (0.60, 2.72) *0.52*	1.05 (0.44, 2.48) *0.91*
Symptoms keep me from working	0.91 (0.58, 1.42) *0.67*	2.54 (1.55, 4.16) *< 0.0001*

^a^Models additionally adjusted for Gender, Insurance, Comorbidities, Visits to the pulmonologist and any doctor, health status and symptoms.

Models evaluating more frequent Internet use (at least weekly versus less frequent) showed that younger age (OR 0.95, 95% CI 0.93, 0.98 per one year increment) was associated with the outcome. Besides age, needs were the factors strongly associated with more frequent Internet use, including perception of diagnosis delay (OR 1.72, 95% CI 1.06, 2.78), feeling treated poorly by the healthcare system (OR 2.46, 95% CI 1.15, 5.24), and that the doctor does not listen (OR 3.14, 95% CI 1.42, 6.95). Additionally, perceiving that the symptoms limit the ability to work was associated with frequent use (OR 2.54, 95% CI 1.55, 4.16).

## Discussion

New information channels, such as the Internet and social media, offer an opportunity for innovative interventions and delivery of health-related education to the chronically ill. COPD patients, with limited mobility, multiple medical needs, coexistent comorbidities, and with a large proportion living in rural areas are good candidates for these interventions. To implement those interventions, preliminary information about patients’ access to information resources and their patterns of use is necessary. Our analysis of a survey of a large convenience sample of COPD patients reveals that there is a significant proportion of the population who does not have Internet access, and that among those with access, there are different patterns of Internet use. These differences in Internet access and use are related to socioeconomic factors, but also to patients’ perceptions of their needs, experience, and levels of satisfaction with the healthcare system. COPD participants without Internet access tended to be older and were more likely to be socioeconomically disadvantaged, while those with access who used the Internet more frequently tended to report greater perceived needs and less satisfaction with the care they had received. Interestingly, no statistically significant differences in markers of disease symptoms or markers of disease severity were found between those who reported Internet access vs. those who did not, with the exception that more frequent Internet users tended to report exacerbations. Additionally, both lack of Internet access and infrequent use of the Internet were also markers of infrequent use of other sources of COPD-related information.

More than one-third of the participants who responded to this survey reported no Internet access. This figure is similar to the 38% figure reported by the Pew Internet Survey of adults living with chronic diseases [13] for the general population in 2008, but lower than the 43.6% figure reported among cancer survivors in a national sample [18], and the 48.5% figure reported by Fashner in a survey of primary care outpatients [19]. The Pew Internet Survey pooled data for patients classified as suffering “lung conditions” reported that 68% had Internet access [13], which is similar to our result among COPD patients. Older age was found to be associated not only with a lack of Internet access but also with less frequent Internet use. This finding (which is similar to that of other surveys of the general population [11,18,20]) can be partially explained by the decline in cognitive and technological readiness present in the elderly, which could affect their performance on tasks requiring more intensive technology use [21,22]. Advanced age is associated with the existence of other barriers such as income and general health, and our data provides support for the existence of a relationship between a person’s income level, educational attainment, and insurance status with both Internet access and the frequency of use. This state of affairs is frequently referred to as the “digital divide” [20,23].

Cardiovascular disease remains the main cause of mortality among COPD patients [24], while mobility-related diseases are strongly related with patients’ perceptions regarding their QOL [25]. Our finding of more cardiovascular and mobility-related comorbidities in those without Internet access is concerning, in conjunction with economic differences, which points toward a disparity in access that targets the most vulnerable patients with COPD. The gap in access may reflect the same socioeconomic factors found in the general population, as well as the additional barrier created by the presence of multiple comorbidities [13].

The finding of a different pattern of Internet use among those with Internet access, with less than one-third being frequent users, is consistent with other surveys [18]. The current analysis also provides evidence of a bivariate association between self-reported anxiety and depression and more frequent Internet use. Other bivariate associations with frequent Internet use included the presence of more perceived needs and less satisfaction with one’s interactions with the healthcare system. Multivariate models identified most of the descriptors of perceived needs (e.g., feeling that one had insufficient time with the doctor, feeling treated poorly, believing that one’s diagnosis was delayed, feeling that one’s physician is not a good listener) as explanatory variables of frequent Internet use.

A challenge to the introduction of new technology is being able to understand factors influencing adoption in the target population. Our findings support the translation of theoretical models used in the communication sciences to the healthcare research and practice demonstrating an association between increased information seeking and motivating factors such as the desire to reduce uncertainty, anxiety, and dread with a need to bolster self-efficacy. Clark et al. have shown similar patterns among breast cancer survivors [26]. The desire to regain a sense of control through the information seeking process as shown by Wong et al. [27] could explain why the more dissatisfied patients in our cohort tended to be more frequent users of the Internet. Models suggest that information seeking is an evolving process that is responsive to changing disease status [28], and differences in disease severity should translate into different behaviors. However, we found an association between frequent Internet use and more exacerbations, but not with other common descriptors of symptoms, disease severity or medication prescriptions. One possible explanation for our findings is that acute exacerbations are probably among the most stressful events in the course of a chronic disease, and increased information seeking is often a response made in an attempt to cope with greater uncertainty and a higher levels of stress generated by the event [29]. The association between poor reported QOL scores and more frequent Internet use may probably reflect the impact of exacerbations on QOL. In contrast, other authors have reported more frequent Internet use among women from the general population and an association with better perceived health [30].

The higher frequency of Internet use by patients who describe less satisfactory interactions with the healthcare system could indicate that patients use the Internet to fill knowledge and orientation gaps generated by an unsatisfactory interaction with the healthcare system. At the same time, these data highlight the potential risks the unsatisfied information seeker maybe exposed to if the information gathered is not reliable .The current survey was not designed to evaluate what type and quality of information was obtained by patients from the Internet and, therefore, can’t answer this question. Additional research in this area will be required. Frequent Internet users were also more frequent users of other health-related information sources (including those that have been judged by some study participants as more helpful, like books and patient organizations [31]), and this process of “redundancy of sources” could actually be protective resulting in better educational opportunities. The net effect is that, even for the unsatisfied population, the desire to obtain information is an appropriate coping skill that needs to be systematically encouraged [32]. In summary, our findings support the conceptual model of information seeking predictions’ that among patients with Internet access, activating mechanisms present after the diagnosis of COPD (more perceived needs and exacerbations) influence the frequency of information seeking on the Internet. Identifying patients with these characteristics is important, as information-avid subjects are probably more willing to engage in clinical decision-making [33].

The finding that lack of Internet access and less frequent Internet use are strongly related to less use of other information and educational resources, with the exception of television and cable services, is not unexpected [34,35], but is concerning, as the sources used by persons without Internet access may be easily available but less helpful or of lower educational content [31]. Wilson’s (1996) Model of Information Behavior describes that there are different types of information seeking, ranging from passive attention to ongoing search. Our findings agree with the model, and prove the presence of a gradient of involvement in the information seeking process, with less frequent Internet users and non-users likely relying on passive diffusion of information, and probably less engagement in the self-care process.

The current analysis uses data from a survey with low response rate, whose population was selected in ways that could bias participation toward patients with more severe cases of COPD (i.e., high frequency of oxygen use and use of oral steroids, as they are selected from participants in support groups or already registered with an oxygen supplier company). The high frequency of participants self-described as of White race may also limit the extrapolation of our findings to the general population. It is also expected that the penetration of the Internet among the chronically ill continue to change since the survey was applied, as it has happened already for the general population (an increment of 10% in Internet use at home between 2007–2011, according to the Census Bureau); however, in the absence of longitudinal data on this question, future research should test if there is change in the frequency of use among COPD patients, and if it is paralleled by what happens in the general population. The strengths of our study include the fact that the survey was based on a large nationwide sample, along with the comprehensive collection of information on respondents’ socio-demographic information and healthcare experiences. Information technology is a rapidly evolving field, and there is always a risk for collected data to become dated rapidly, but even with these caveats this is the first evaluation of the specific characteristics determining Internet access and use by COPD patients in the community, outside of a clinical or implementation trial [36].

**Figure 3:**
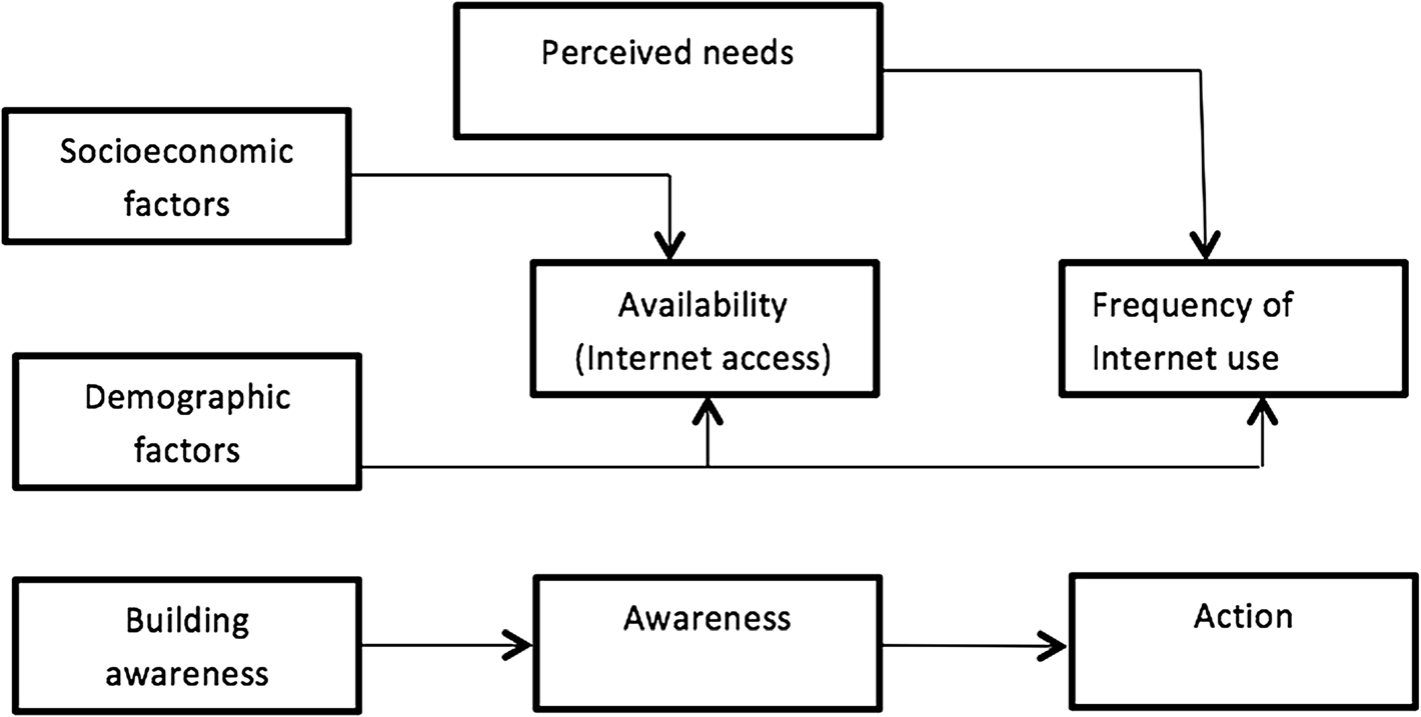
**Conceptual model of the relationships between COPD patient characteristics, perceived needs, and Internet access.** We hypothesize that patient demographic and socioeconomic factors are related with the availability of the resource (access to the Internet); while frequency of Internet use is related with the patient’s perceived needs. The simplified model’s relationships are based on a modification of Wilson’s (1996) Model of Information Behavior, as described in Figure 1.

Finally, our findings align relatively well with Wilson’s (1996) Model of Information Behavior, which could be used to design future Internet-mediated interventions. A simplified model (Figure 3) informed by our analysis describes how after an initial stress (having a diagnosis of COPD) specific demographic and socioeconomic factors predict Internet access and that among those with access, activating mechanisms based on the risks and potential rewards (perceived needs) help to explain the frequency of Internet use. The model could also be used to design and test interventions to increase the use of information technology among chronic patients.

## Conclusions

We identified significant differences between participants based on Internet access and frequency of use. The differences we found provide support for our hypotheses that Internet access, similar to other social engagement opportunities, is related to socioeconomic factors, while frequency of Internet use is associated with patients’ perceived needs, experience with the healthcare system, and history of COPD-related exacerbations. Lack of Internet access and infrequent Internet use were found to also be markers of less use of other COPD-related information sources. To the best of our knowledge, this is the first survey analysis to focus on Internet access and use among COPD patients. Recognizing and working to diminish the “digital divide” among these subpopulations of COPD patients and striving to better identify and fill their information needs could provide new opportunities for improved patient education and increased patient participation in their own healthcare and in Internet-mediated outreach and research.

## Competing interests

The authors declare that they have no competing interests.

## Authors’ contributions

CHM, BLSJ, CAP, BR, JB, FJM, CRR, MKH were involved in the conception and design of the study. CAP, BR, JB, FJM were involved in study data collection. CHM, BLSJ, CAP, BR, JB, FJM, CRR, MKH conducted study analyses. All authors read and approved the final manuscript.

## Authors’ information

Co-senior authors: Caroline R Richardson and MeiLan K Han.

## Pre-publication history

The pre-publication history for this paper can be accessed here:

http://www.biomedcentral.com/1471-2466/14/66/prepub

## Supplementary Material

Additional file 1 Needs assessment of patients with lung disease in the United States. Click here for file
