# Supplementary material for: Internet access and use by COPD patients in the National Emphysema/COPD Association Survey
Source: BMC Pulm Med. 2014 Apr 22;14:66. doi: 10.1186/1471-2466-14-66 (PMC4021217; doi:10.1186/1471-2466-14-66)
Supplement: Additional file 1 — Needs assessment of patients with lung disease in the United States. [file 1471-2466-14-66-S1.pdf]

NECA
National Emphysema/COPD Association

INNOVATIVE HEALTH SOLUTIONS

NEEDS ASSESSMENT OF PATIENTS WITH LUNG DISEASE IN THE UNITED STATES

We'll begin with a few questions about your medical history.

1. Which of the following conditions, if any, have you ever been diagnosed with? *Check all that apply.*

Lung disease

- ❑ Chronic Bronchitis
- ❑ Emphysema
- ❑ COPD
- ❑ Alpha-1 Antitrypsin Deficiency
- ❑ Asthma
- ❑ Other chronic lung disease (please specify): ____________________

Other conditions

- ❑ Anxiety
- ❑ Arthritis
- ❑ Breast Cancer
- ❑ Depression
- ❑ Diabetes
- ❑ Heart Disease
- ❑ High Blood Pressure
- ❑ Lung Cancer
- ❑ Migraines
- ❑ Obesity
- ❑ Osteoporosis
- ❑ Parkinson's Disease
- ❑ Prostate Cancer
- ❑ Renal Failure
- ❑ Sleep Disorder
- ❑ Stroke
- ❑ Other (please specify): ____________________

2. At what age were you first diagnosed with a chronic lung disorder?

________(age)

3. Have any of these relatives ever been diagnosed with emphysema, chronic bronchitis or COPD? *Check all that apply.*

- ❑ Grandfather or grandmother
- ❑ Father or mother
- ❑ Uncle or aunt
- ❑ Brother or sister
- ❑ Nephew, niece or cousin
- ❑ None of these

4. Has there been any three month period during the past year when you had the following symptoms every day or most days a week? *Check all that apply.*

- ❑ Coughed
- ❑ Brought up phlegm or sputum
- ❑ Had shortness of breath
- ❑ Been awakened at night by coughing, wheezing or shortness of breath
- ❑ None of these

5. At what age did you first have any of these symptoms every day or most days a week for several months?

________(age)

6(a). Do you believe you received a formal diagnosis of chronic lung disease shortly after you first exhibited symptoms? *Choose one.*

- ❍ Yes, my doctor(s) was(were) on top of my situation [*Please advance to Question 7*]
- ❍ No, I should have received a formal diagnosis sooner than I did
- ❍ Not sure

6(b). Were these symptoms of lung disease first diagnosed as one of the following? *Choose one.*

- ❍ Anxiety
- ❍ Asthma
- ❍ Bronchitis
- ❍ Other (please specify): ____________
- ❍ Not sure

7(a). Have you had any occupational or environmental exposures that may have caused or contributed to your condition?

- ❍ No
- ❍ Yes

7(b). If "yes," please describe the specific occupational or environmental exposures:

Now we'd like to ask a few questions about your current state of health.

8. How would you describe your overall health status at the moment?

- ❍ Excellent
- ❍ Very good
- ❍ Good
- ❍ Fair
- ❍ Poor
- ❍ Very poor

9. How severe are your current COPD symptoms?

- ❍ No symptoms
- ❍ Mild
- ❍ Moderate
- ❍ Quite severe
- ❍ Very severe

10. Which of the following best describes how breathless you get these days? *Choose one.*

- ❍ I am too breathless to leave the house
- ❍ I have to stop for breath after walking a few minutes on level ground
- ❍ I have to stop for breath when walking on level ground at my own pace
- ❍ I walk slower than most people my age
- ❍ I get breathless when hurrying on level ground or walking up a slight incline
- ❍ I only get breathless after strenuous exercise
- ❍ None of these

11(a). Does your lung condition… *Check all that apply.*

- ❑ Keep you from working
- ❑ Limit amount or type of work you can do
- ❑ Limit activities other than working [*Please answer Question 11(b)*]
- ❑ Not limit your activity

11(b). In what other ways does your condition limit your activities? *Check all that apply.*

- ❑ Bathing/showering
- ❑ Bending over/tying shoes
- ❑ Climbing stairs
- ❑ Dressing
- ❑ Driving
- ❑ Eating (larger meals)
- ❑ Going from bed or chair
- ❑ Grooming
- ❑ Housework (incl. dusting, sweeping, vacuuming, laundry, etc.)
- ❑ Intimacy
- ❑ Long conversations
- ❑ Other (please specify): ___________________
- ❑ Meal preparation
- ❑ Outdoor activities /gardening
- ❑ Paying bills
- ❑ Shopping
- ❑ Sleeping
- ❑ Social interactions /visiting friends or family
- ❑ Toilet use
- ❑ Travel
- ❑ Walking

12. During the past 12 months, as a result of your lung condition have you been/had... *Check all that apply.*

- ❑ Hospitalized overnight or longer
- ❑ A hospital emergency room/urgent care visit
- ❑ Other emergency doctor visits
- ❑ None of these

Now we'd like to ask about your visits to doctors to treat your lung disease and any other health conditions.

---

13. How many times have you seen any doctor about your health in the past 12 months?

_____(# of times)

14. How many times have you seen a lung specialist about your breathing or lung condition in the past 12 months?

______(# of visits)

15. How many times have you seen any doctor other than a lung specialist about your breathing or lung condition in the past 12 months?

______(# of visits)

16. Which of these doctors is primarily responsible for the management of your breathing or lung condition? *Choose one.*

- ❑ General/Family Practice
- ❑ Internal Medicine
- ❑ Pulmonology/Lung specialist
- ❑ Cardiology/Heart specialist
- ❑ Hepatology/Liver specialist
- ❑ Otolaryngology/ENT
- ❑ Other, please specify _______________
- ❑ No one [*Please advance to Question 19*]

17. Overall, how satisfied are you with this doctor's management and treatment of your breathing or lung condition? *Choose one.*

- ❍ Very satisfied
- ❍ Somewhat satisfied
- ❍ Somewhat dissatisfied
- ❍ Very dissatisfied

18. What are you least satisfied about concerning the management and treatment of your breathing or lung condition? *Select all that apply.*

- ❑ Non-aggressive treatment
- ❑ Questions not answered by doctor(s)
- ❑ Doctor's lack of knowledge about COPD
- ❑ Other (please specify): _______________
- ❑ Other (please specify): _______________
- ❑ Other (please specify): _______________

19. If you have multiple health conditions, which of the following other types of doctors do you see on a regular basis for treatment of your various conditions (other than for your breathing or lung condition)? *Select all that apply.*

- ❑ Cardiologist
- ❑ Primary Care Physician /Internist
- ❑ Psychologist/Psychiatrist
- ❑ Endocrinologist
- ❑ Orthopedist
- ❑ Other (please specify): _____________
- ❑ Immunologist
- ❑ Dermatologist
- ❑ Ear, Nose and Throat specialist
- ❑ Rheumatologist

20. If you answered the previous question, how well coordinated is the care you receive for your breathing or lung condition for each of the following among these multiple doctors? *Choose one response for each row.*

	Very well	Quite well	Moderately well	Some-what well	Not at all well
Diagnostic testing	❍	❍	❍	❍	❍
Prescription medicines	❍	❍	❍	❍	❍
Communication among doctors	❍	❍	❍	❍	❍
Surgery	❍	❍	❍	❍	❍

Now we'd like to ask about your treatment of your lung disease.

21(a). Have you ever had breathing tests for your lungs – where you blow hard into a tube? *Choose one.*

- ❍ Never [*Please advance to Question 22*]
- ❍ Yes

21(b). How often has your lung function been tested by a doctor, a respiratory therapist or a nurse in the past 12 months? *Chose one for each column.*

	Doctor	Respiratory Therapist	Nurse
Every visit	❍	❍	❍
Once a month	❍	❍	❍
Several times a year	❍	❍	❍
Every six months.	❍	❍	❍
Just once	❍	❍	❍
Not in past 12 months	❍	❍	❍

22(a). Have you used oxygen therapy outside a hospital on a regular basis in the past 12 months? *Choose one.*

- ❍ No [*Please advance to Question 23*]
- ❍ Yes

22(b). When do you use oxygen? *Choose one.*

- ❍ Only during the day
- ❍ Only at night
- ❍ Both
- ❍ Other, please specify___________________

22(c). Do you use oxygen as much as prescribed by your doctor? *Choose one.*

- ❍ No
- ❍ Yes [*Please advance to Question 23*]

22(d). Why do you not use oxygen as much as prescribed by your doctor? *Select all that apply.*

- ❑ Expense
- ❑ Fear of appearing "sick"
- ❑ Inconvenience
- ❑ Not allowed in work environment
- ❑ Interferes with work activities/tasks
- ❑ Interferes with household responsibilities including cooking, cleaning, or care giving
- ❑ Other (please specify): _____________

23. What is(are) your preferred way of taking medication(s) for COPD? *Select all that apply.*

- ❑ Pill or tablet
- ❑ Liquid
- ❑ Dry –powdered inhaler (DPI)
- ❑ None
- ❑ Metered dose inhaler (MDI)
- ❑ Injection
- ❑ Nebulizer

24. How often, if at all, have you used oral steroids (taken in pill form) for your respiratory condition in the past 12 months? *Choose one.*

- ❍ All or most of the time
- ❍ Only during exacerbations
- ❍ Never

25. Overall, how effective are your current medications for the treatment of your breathing or lung condition? *Choose one.*

- ❍ Very effective
- ❍ Somewhat effective
- ❍ Somewhat ineffective
- ❍ Very ineffective

26. How likely are you to FILL a prescription given to you by your physician for a COPD medicine you have NEVER taken before? *Choose one.*

- ❍ All of the time *[Please advance to Question 28]*
- ❍ Often
- ❍ Sometimes
- ❍ Rarely
- ❍ Never

27. What are the reason(s) you do not always FILL a prescription for a COPD medicine you have NEVER taken before? *Rank the following possible reasons in order of importance ("1" being the most important reason); skip any reason that is not applicable to you.*

_ Financial/insurance coverage
_ Feel I don't need it
_ Fearful of any side effects
_ Fearful of interactions with other drugs I take
_ Forget
_ Other (please specify): ________________

28. How likely are you to REFILL a prescription for a COPD medicine you have taken before? *Choose one.*

- ❍ All of the time [*Please advance to Question 30(a)*]
- ❍ Often
- ❍ Sometimes
- ❍ Rarely
- ❍ Never

29. What are the reason(s) you do not always REFILL a prescription for a COPD medicine you have taken before? *Rank the following possible reasons in order of importance ("1" being the most important reason); skip any reason that is not applicable to you.*

_ Financial/insurance coverage
_ Feel I don't need it
_ Fearful of any side effects
_ Fearful of interactions with other drugs I take
_ Forget
_ Other (please specify): ________________

30(a). Have you ever stopped taking (or not taken) your COPD medications as prescribed?

- ❍ No [*Please advance to Question 31(a)*]
- ❍ Yes

30(b). What are the reason(s) for having stopped taking (or not taken) your COPD medications as prescribed? *Rank the following possible reasons in order of importance ("1" being the most important reason); skip any reason that is not applicable to you.*

_ Financial/insurance coverage
_ Feel I don't need it
_ Fearful of any side effects
_ Fearful of interactions with other drugs I take
_ Forget
_ Other (please specify): ________________

31(a). Are you aware of any standard treatment guidelines for the care of lung disease and/or COPD?

- ❍ No [*Please advance to Question 32*]
- ❍ Yes

31(b). How did you learn about these standard guidelines? *Select all that apply.*

- ❑ From my respiratory therapist
- ❑ From my caregiver
- ❑ From a support group
- ❑ From a web site (not support group site)
- ❑ From my primary care physician
- ❑ From a pulmonary specialist
- ❑ Other (please specify): ________________

32. Which of the following statements best describes your attitudes toward pulmonary rehabilitation programs for patients with chronic lung diseases? *Check all that apply.*

- ❑ I am not aware of such programs
- ❑ I don't believe they are very helpful
- ❑ I am not certain how helpful they are
- ❑ They are helpful, but I don't have access
- ❑ I have been referred, but not used them
- ❑ I have participated in such programs

33. If you have multiple health conditions, which of the following three statements best describes your prioritization of your multiple health conditions. *Check one response.*

- ❍ My COPD/lung disease is the most serious of my various health conditions, and it is the #1 priority in my various treatment regimens.
- ❍ My COPD/lung disease is one of several health conditions and is secondary to at least one of my other health conditions.
- ❍ I do not prioritize my conditions; they're equally important. [*Please advance to Question 35*]

34. How do you prioritize the treatment of your multiple health conditions?

35. Please indicate your level of agreement with each of the following statements using the five-point scale provided.

	Agree Strongly	Agree	Neither Agree nor Disagree	Disagree	Disagree Strongly
My doctor is sympathetic to my condition	❍	❍	❍	❍	❍
As a smoker/former smoker, I am treated poorly by the medical community	❍	❍	❍	❍	❍
My time with my doctor is sufficient	❍	❍	❍	❍	❍
My doctor does not listen to me	❍	❍	❍	❍	❍
I am able to reach my doctor when I need care	❍	❍	❍	❍	❍
There are no pulmonary rehabilitation clinics available in my area	❍	❍	❍	❍	❍

	Agree Strongly	Agree	Neither Agree nor Disagree	Disagree	Disagree Strongly
I am able to get an appointment with my doctors when I need one	❍	❍	❍	❍	❍
I have no transportation to my doctor(s)	❍	❍	❍	❍	❍

Please tell us about how you pay for your treatment of your lung disease.

36. What is(are) the current source(s) of your health care coverage (including through other family members)? *Check all that apply.*

- ❑ Insurance through employer/work
- ❑ Other group insurance
- ❑ Individual policy
- ❑ COBRA
- ❑ Medicare
- ❑ Medicaid/MediCal
- ❑ State/county health agency
- ❑ Veteran's Administration
- ❑ Other (please specify): ___________________
- ❑ No insurance coverage

37. Has your insurance coverage acted as a barrier to any of the following? *Check all that apply.*

- ❑ Access to home oxygen therapy
- ❑ Access to other medical specialists
- ❑ Access to pulmonary rehabilitation programs
- ❑ Access to respiratory therapists
- ❑ Antibiotic therapy
- ❑ Prescription drugs for symptom relief
- ❑ Prescription drugs for underlying condition
- ❑ Referrals to lung specialist
- ❑ Regular lung testing
- ❑ Routine visits to primary care doctors
- ❑ None of these

38. Beyond insurance, which of the following, if any, have you had to use to pay for treatment of the lung disease (including medicines, therapy or hospitalizations)? *Check all that apply.*

- ❑ Personal savings
- ❑ Retirement accounts
- ❑ Sold stocks or bonds
- ❑ Mortgaged the house
- ❑ Obtained a loan
- ❑ Worked overtime
- ❑ Found a second job
- ❑ Borrowed on life insurance
- ❑ Charity
- ❑ Other (please specify): ___________________
- ❑ None of these

How do you obtain information about your lung disease?

39. How well informed do you feel you are about your condition and treatment? *Choose one.*

- ❍ Very well informed
- ❍ Adequately informed
- ❍ Less than adequately informed
- ❍ Very poorly informed

40. Which of the following have you used to get information about your condition and treatment in the past 12 months? *Check all that apply.*

- ❑ Doctors
- ❑ Nurses
- ❑ Respiratory therapists
- ❑ Caregivers
- ❑ Patient organizations
- ❑ Other patients
- ❑ Books or magazines
- ❑ Pamphlets/brochures
- ❑ Television or cable
- ❑ Online support groups
- ❑ Other Internet sites
- ❑ None of these
- ❑ Other (please specify): ___________________

41. Which of the following, if any, are you aware of with regard to your lung disease/COPD? *Check all that apply.*

- ❑ How to avoid lung infections
- ❑ How to prevent your condition from worsening
- ❑ Actions to take when symptoms worsen
- ❑ Recognizing signs when emergency assistance is required
- ❑ Pulmonary rehabilitation
- ❑ Spirometry
- ❑ Arterial blood gas
- ❑ Pulmonary function tests
- ❑ Oximetry
- ❑ Standard treatment guidelines for the care of lung disease and/or COPD

42. In the past 12 months, which of the following material have you received from patient organizations dealing with chronic lung disease? *Check all that apply.*

- ❑ Newsletters
- ❑ Patient education materials
- ❑ Fund solicitations
- ❑ Medical updates
- ❑ Product warnings or recalls
- ❑ Invitations to meetings
- ❑ Invitations to support groups
- ❑ Patient surveys
- ❑ Other (please specify): ___________________
- ❑ None of these

43(a). In the past 12 months, have you interacted with patient organizations dealing with chronic lung disease?

❍ No [*Please advance to Question 44*]
❍ Yes

43(b). In which ways of the following ways, if any, have you interacted with such patient organizations in the past 12 months? *Check all that apply.*

❑ Written for information or assistance
❑ Called for information or assistance
❑ E-mailed for information or assistance
❑ Contacted through web site
❑ Participated in support group
❑ Participated in local meeting
❑ Participated in national meeting
❑ Participated in research project
❑ Other (please specify): ________________
❑ None of these

44. How important is it to you, personally, to see each of the following types of activities promoted for patients with chronic lung disease? *Choose one for each row.*

	Not Important	Slightly Important	Important	Fairly Important	Very Important
Access to treatment	❍	❍	❍	❍	❍
Case management	❍	❍	❍	❍	❍
Clinical research studies	❍	❍	❍	❍	❍
Development of new therapies	❍	❍	❍	❍	❍
Patient/family educational materials	❍	❍	❍	❍	❍
Peer counseling	❍	❍	❍	❍	❍
Physician education	❍	❍	❍	❍	❍
Public awareness	❍	❍	❍	❍	❍
Public policy advocacy	❍	❍	❍	❍	❍
Pulmonary rehab.	❍	❍	❍	❍	❍
Respiratory therapist education	❍	❍	❍	❍	❍
Nurse therapist education	❍	❍	❍	❍	❍
Reimbursement advocacy	❍	❍	❍	❍	❍
Research funding	❍	❍	❍	❍	❍
Scientific meetings/ conferences	❍	❍	❍	❍	❍
Screening & detection	❍	❍	❍	❍	❍
Support groups	❍	❍	❍	❍	❍
Telephone info & referrals	❍	❍	❍	❍	❍
Transplant info & advocacy	❍	❍	❍	❍	❍
Other	❍	❍	❍	❍	❍

If "Other," please specify: ______________________

45. For any activity you checked "Very important" or "Important" in the prior question, which three are the most important? *Select three.*

❑ Access to treatment
❑ Case management
❑ Clinical research studies
❑ Development of new therapies
❑ Patient/family educational materials
❑ Peer counseling
❑ Physician education
❑ Public awareness
❑ Public policy advocacy
❑ Pulmonary rehabilitation
❑ Respiratory therapist education
❑ Nurse therapist education
❑ Reimbursement advocacy
❑ Research funding
❑ Scientific meetings/conferences
❑ Screening and detection
❑ Support groups
❑ Telephone info & referrals
❑ Transplant info & advocacy
❑ Other

46. Overall, how would you rate the job that patient organizations have done in meeting your needs as a patient with a chronic lung disease? *Choose one.*

❍ Excellent
❍ Very good
❍ Adequate
❍ Less than adequate
❍ Poor

47. How often do you use the <u>Internet</u> to get information about your condition or its treatment? *Choose one.*

❍ At least weekly
❍ At least monthly
❍ A few times a year
❍ Rarely/never
❍ No Internet access

<u>Please read this carefully</u>

On the following page you will find some statements that have been made by people who have Chronic Obstructive Pulmonary Disease (COPD)/breathing problems.

Thinking about your COPD/breathing problems, please read each statement carefully and put a tick in the box ☑ next to the response that best applies to your life right now.

Please choose the response that best applies to your life right now.

The statements below ask about your experience of having COPD/breathing problems. Thinking about your COPD/breathing problems please read each statement carefully and put a tick ☑ next to the response that best applies to your life right now.

	True	Not True
1. My illness limits the places I can go	❑	❑
2. I get frustrated easily	❑	❑
3. I can't do things on the spur of the moment	❑	❑
4. I feel like a prisoner in my own home	❑	❑
5. I worry that I stop people doing what they want to do	❑	❑
6. My illness controls me	❑	❑
7. I have to plan even the most simple tasks carefully	❑	❑
8. My breathing makes me self conscious	❑	❑
9. I have to pace myself	❑	❑
10. I feel dependent on others	❑	❑
11. Going out for a day is too much for me	❑	❑
12. My illness frightens me	❑	❑

Please remember to put a tick in only one of the alternative responses for each of the statements.

	True	Not True
13. I can't make long-term plans	❑	❑
14. I don't feel like socializing	❑	❑
15. I can't put a great deal of effort into anything	❑	❑
16. My self-confidence is affected	❑	❑
17. It is with me every day of my life	❑	❑
18. I rarely go out of the house	❑	❑

Please read each statement carefully and decide whether it applies to your life right now .

	True	Not True
19. My physical limitations get me down	❑	❑
20. My illness affects my close relationships	❑	❑
21. My illness restricts my social activities	❑	❑
22. My illness determines what I can do	❑	❑

Please check all the responses to make sure that you have answered every statement.

Finally, please answer these demographic questions so that we might group your responses with others like you.

What is your YEAR of birth?

19__

What is your gender?

- ❍ Male
- ❍ Female

What is your level of education?

- ❍ Some high school
- ❍ Graduated high school
- ❍ Some college
- ❍ Graduated college
- ❍ Graduate/professional degree

In what US state do you live?

____

Do you live in an urban, suburban or rural area?

- ❍ Urban
- ❍ Suburban
- ❍ Rural

What is your annual household income?

- ❍ Under $20,000
- ❍ $20,000 - $29,999
- ❍ $30,000 - $49,999
- ❍ $50,000 - $74,999
- ❍ $75,000 - $99,999
- ❍ $100,000 - $150,000
- ❍ Over $150,000

Which of the following racial designations in the US Census best fits you?

- ❍ American Indian or Alaska Native
- ❍ Asian
- ❍ Black or African American
- ❍ Native Hawaiian or Other Pacific Islander
- ❍ White
- ❍ Some other race (please specify): ____________

Do you have any other comments?

Thank You!

If you would like to receive a copy of this study's findings or would like to participate in future COPD research studies, please provide your contact information on the separate form.

NO POSTAGE
NECESSARY
IF MAILED
IN THE
UNITED STATES

BUSINESS REPLY MAIL

FIRST-CLASS MAIL PERMIT NO. 32 WESTPORT CT

POSTAGE WILL BE PAID BY ADDRESSEE

NEEDS ASSESSMENT SURVEY
NATIONAL EMPHYSEMA / COPD ASSOC
PO BOX 2814
WESTPORT CT 06880-9948

After completing the survey, please fold this page in half along the dotted line, tape the two sides together and drop it off in the US mail.

Postage is pre-paid.

Thank you again for your participation.
